# Application for Ecological Restoration of Contaminated Soil: Phytoremediation

**DOI:** 10.3390/ijerph192013124

**Published:** 2022-10-12

**Authors:** Rongkui Su, Yangyang Wang, Shunhong Huang, Runhua Chen, Jun Wang

**Affiliations:** 1 College of Environmental Science and Engineering, Central South University of Forestry & Technology, Changsha 410004, China; 2 PowerChina Zhongnan Engineering Corporation Limited, Changsha 410004, China; 3 College of Geography and Environmental Science, Henan University, Kaifeng 475004, China

Nowadays, with the rapid development of industry and agriculture, heavy metal pollution is becoming more and more serious, mainly deriving from natural and man-made sources [1,2,3]. Natural sources include volcanic eruptions, forest fires, fossil fuels, etc., while human sources include agricultural fertilizer, mining, smelting plants, etc. [4,5]. Metal from natural sources does little harm to the ecological environment, and the heavy metal pollution produced artificially is particularly serious [6,7,8]. Almost all countries in the world are affected by heavy metal pollution [9,10]. It is reported that there are more than ten million polluted areas in the world, of which more than 50% are affected by heavy metal pollution [10]. In the United States, there are about 600,000 sites polluted by heavy metals, and 1.4 million sites are affected by heavy metals in Western Europe [11,12,13]. One-sixth of China’s cultivated land is polluted by heavy metal, and more than 40% of the cultivated land is degraded to varying degrees due to erosion and desertification. Heavy metals affect the composition, structure, function, biomass and diversity of soil microbial communities [14,15,16,17]. Additionally, they can enter the human body after the biological amplification of the food chain, thereby endangering human health [18]. The long-term consumption of food contaminated by heavy metals will lead to mutations in human genes and metabolic enzymes [19], increase the incidence of diseases in the esophagus, blood, kidney, nervous system, cardio cerebrovascular and urinary system, and induce cancer, organ failure, etc. [20,21]. Compared with organic pollutants [22,23,24,25,26], heavy metals are not easily biodegraded and can persist in nature for more than 20 years, leading to the continuous accumulation of heavy metals in the environment, posing a serious threat to human and ecological security [27,28]. Therefore, the treatment of heavy metal-contaminated soil is extremely urgent.

The remediation methods of heavy metal-contaminated soil mainly include physical remediation, chemical remediation and biological remediation [29]. Physical remediation methods include soil replacement, vitrification, solidification, etc. Physical remediation methods have the advantages of wide application, thoroughness and efficiency, but there are shortcomings such as requiring a large amount of work, damage to soil structure, high cost, high energy consumption, etc. [30,31,32]. Chemical remediation mainly includes oxidation, reduction, adsorption, precipitation, polymerization, complexation, etc. It has the advantages of a short cycle and can be used to treat various pollutants, but chemical remediation produces secondary pollutants, disturbs the physical and chemical properties of soil, and changes the original microbial community [33,34,35,36]. Bioremediation includes microbial remediation, animal remediation and phytoremediation [37,38,39,40]. Microbial remediation has the advantages of low cost and little disturbance to the soil, but it is only applicable to the soil polluted by low concentrations of heavy metal and vulnerable to environmental impact [41]. Animal remediation methods can improve soil and enhance soil fertility, but animals that accumulate a large amount of heavy metals in their bodies will continue to migrate, and so they need to be properly disposed of [42]. The above methods have achieved some promising results. However, based on the obvious shortcomings, it is difficult to scale up their application. Therefore, it is extremely important to develop sustainable heavy metal soil remediation technology.

In recent years, phytoremediation has been widely studied as an economic, effective and green technology. Phytoremediation has become a research hotspot since Chaney et al. proposed using hyperaccumulative plants to restore heavy metal-contaminated soil [43,44]. Phytoremediation is a green, economic and environmental remediation technology for heavy metal-contaminated soil, which uses the absorption, volatilization, transformation and degradation of plants (“hyperaccumulator”) with strong enrichment capacity for heavy metals and their rhizosphere microbial system to absorb or stabilize heavy metals in soil [45,46,47,48]. The principle of phytoremediation mainly includes plant extraction, plant volatilization, plant stability, plant degradation, etc. [6,49,50,51,52]. Plant extraction refers to using technology that uses plant roots to absorb heavy metals from the soil and transfer and accumulate them to aboveground plants (buds, leaves, etc.). Plant extraction is divided into natural plant extraction technology and chemically induced plant extraction technology [45,53]. Compared with other phytoremediation technologies, it is more suitable for commercial applications [54]. Plant volatilization is closely related to plant absorption. The principle of plant volatilization technology is that plants use root exudates or rhizosphere microorganisms to absorb and accumulate pollutants in the soil, transform them into less toxic and volatile forms, and finally release them into the atmosphere through plant transpiration. At present, plant volatilization technology is used primarily to restore the soil polluted by Hg, Se and As [45]. Plant stability, also known as plant fixation, mainly refers to the accumulation, adsorption and precipitation of heavy metals in the soil by plant roots, thus reducing their mobility and bioavailability and limiting their leaching into groundwater and the food chain [55,56,57,58,59]. Another function of plant stability is to reduce soil erosion. Plant stabilization technology is widely used to restore abandoned mining areas polluted by Zn, Pb, Cd, Mn, Cu, Cr, Fe, As, Ni and other metals, and plants have specificity for the stability of these metals [45,50,51,56]. For instance, reed and cattail stabilize As and Hg, and have little effect on other metals [60]. Plant degradation, also known as plant transformation, is based on the principle that plants degrade or transform pollutants into an environmentally friendly state through root exudates. Plant degradation mainly targets the most complex organic molecules and a few heavy metal pollutants [6].

The efficiency of the phytoremediation of heavy metal-contaminated soil mainly depends on plant biomass and its ability to accumulate heavy metals [61,62]. However, wild hyperaccumulators generally have the defects of a long growth cycle and small biomass, and the types, forms and contents of heavy metals in soil will affect the tolerance and remediation efficiency of plants, which seriously restricts the application of phytoremediation technology in the remediation of heavy metal-contaminated soil [61]. In addition, it is unlikely that a single piece of phytoremediation technology will restore heavy metal-contaminated soil on its own, so it needs to be combined with other remediation technologies, such as microbial phytoremediation technology, chemical phytoremediation technology, physically assisted phytoremediation technology, etc. [63,64,65,66,67,68]. Soil microorganisms often co-exist with plants. The nutrient-rich exudates in the rhizosphere of plants can promote the growth of microbial communities. Microbes can provide an ideal soil environment for plants, enhance plant resistance, assist plants to absorb water and minerals from the soil, stimulate plants to secrete hormones, and shorten the plant growth cycle [54,63,69]. Chemical methods combined with phytoremediation mainly use chemical reagents to assist plants to extract heavy metals from soil or enhance the stability of some plants to heavy metals [64,65,66]. Phytoremediation assisted by physical methods can remedy the limitations of phytoremediation. For instance, the combination of electric restores and phytoremediation is one of the most commonly used methods [67,68].

Soil heavy metal pollution is a major problem worldwide. Compared with physical and chemical remediation methods, phytoremediation is a mild, relatively cheap and ecologically safe technology with huge potential. Looking forward, the following points deserve more attention: (1) Using molecular biology and transgenic engineering technology, we should screen and cultivate local genetic engineering bacteria and genetic engineering plants under heavy metal stress, and transfer target functional genes to plants, so as to improve the relevant remediation capacity of heavy metal-contaminated environments and reduce the disturbance to the contaminated soil ecosystem. (2) There is little concern about the remediation of heavy metal-contaminated soil under organic compound pollution, and the phytoremediation technology of organic–inorganic compound pollutants will be a hotspot in the future. (3) We need to find more efficient and safer microbial plants, chemical plants and other joint remediation technologies. (4) More and more attention will be given to the woody plant remediation technology with large biomass, great economic value and environmental value. (5) The application of artificial intelligence and machine learning for the environment will be more and more prevalent. Artificial intelligence and machine learning have huge application prospects in the research of heavy metal pollution remediation through plants, gene improvement and site remediation. (6) In future research, the medium scale remediation test of actual heavy metal pollution should carry out site construction to accelerate the transition of phytoremediation technology from a simulation experiment to practical application.

This Special Issue was created to promote researchers in this field, presenting their thoughts, ideas, and discoveries of phytoremediation in environmental applications. Thank you to everyone who wants to or can contribute to this Special Issue.
